# Exploring expected values of topological indices of random cyclodecane chains for chemical insights

**DOI:** 10.1038/s41598-024-60484-x

**Published:** 2024-05-02

**Authors:** Bai Chunsong, Anisa Naeem, Shamaila Yousaf, Adnan Aslam, Fairouz Tchier, Abudulai Issa

**Affiliations:** 1 School of Finance and Mathematics, Huainan Normal University, Huainan, 232038 China; 2 Department of Mathematics, University of Gujrat, Hafiz Hayat Campus, Gujrat, Pakistan; 3 Department of Natural Sciences and Humanities, University of Engineering and Technology, (RCET), Lahore , Pakistan; 4 Mathematics Department, College of Science, King Saud University, P.O. Box 22452, 11495 Riyadh, Saudi Arabia; 5 Department of Mathematics, University of Lome, P. O. Box 1515, Lome, Togo

**Keywords:** Chemical graph theory, Topological indices, Cyclodecane chains, Expected values, Analytical chemistry, Theoretical chemistry

## Abstract

Chemical graph theory has made a significant contribution to understand the chemical compound properties in the modern era of chemical science. At present, calculation of the topological indices is one of most important area of research in the field of chemical graph theory. Cyclodecane is a cyclic hydrocarbon with the chemical formula $C_{10}H_{20}$. It consists of a ring of ten carbon atoms bonded together in a cyclical structure. Cyclodecane chains can be part of larger molecules or polymers, where multiple cyclodecane rings are connected together. These molecules can have various applications in chemistry, materials science, and pharmaceuticals. This article aims to determine expected values of some connectivity based topological indices of random cyclodecane chains, containing saturated hydrocarbons with at least two rings. It also compares these descriptors using explicit formulae, numerical tables and present graphical profiles of these comparisons.

## Introduction

Chemical graph theory is a branch of graph theory that focuses on the study of graphs to model and understand molecular structures and chemical reactions. In this context, atoms are represented as vertices (nodes), and chemical bonds are represented as edges connecting these vertices. It provides a powerful framework for understanding molecular structures, properties, and reactions, and plays a central role in many areas of chemistry, biochemistry, and materials science. Chemical graph theory is used to predict molecular structures based on connectivity information. Algorithms such as the Morgan algorithm or the famous Wiener index can be used to generate molecular structures or predict properties like molecular shape, size, and symmetry. QSAR studies correlate the chemical structure of molecules with their biological activity or other properties. Graph-based descriptors derived from chemical graphs, such as topological indices, connectivity indices, and molecular fingerprints, are utilized to quantify structural features and predict biological activity.

A topological index is a numerical value assigned to a molecular structure based solely on its topology, or connectivity pattern, without considering bond lengths or angles. These indices are used in chemical graph theory and quantitative structure-activity relationship (QSAR) studies to correlate molecular structure with physical, chemical, or biological properties. Topological indices provide a simplified representation of molecular structure, facilitating the comparison of molecules and the prediction of their properties. There are many degree and distance based topological indices introduced in literature but some of them are better because of their correlation with chemical properties such as high boiling point, strain energy and stability. The degree based topological indices link specific physicochemical characteristics of several chemical substances. For more detail on various topological indices, see^1–11^. The name molecular descriptor was introduced for the Z-index^12^. For details, see^13–15^. The quantitative structure property relationship (*QSPR*) and the quantitative structure activity relationship (*QSAR*) are two areas in which topological indices have particularly vital role in mathematical chemistry^16,17^.

A graph $\Upsilon$ is made up of two finite sets, vertices and edges. The degree of the vertex $\upsilon$ is the number of edges that incident at vertex $\upsilon$ in $\Upsilon$ and it is denoted by the symbol $d(\upsilon )$. For basic terminologies related to graph theory, the readers can see^10^. The Randić index^18^, first introduced by Milan Randić in 1975, measures molecular branching of chemical compounds in graph theory. The mathematical formula of Randić index is

1 $$\begin{aligned} R(\Upsilon )&=\sum _{\upsilon ,\nu \in E(\Upsilon )}\frac{1}{\sqrt{d(\upsilon )d(\nu )}}. \end{aligned}$$

It is useful in quantitative structure-activity relationship (QSAR) studies in chemistry, correlated with properties like boiling points, enthalpies, and molecular weights. It captures information about molecular structure branching and connectivity, making it a valuable tool in chemical graph theory and molecular graph analysis. Details on these applications can be found in the books^19–21^. The General Randić index^22^, also known as the General Randić connectivity index, is an extension of the Randić index, focusing on the molecular branching of chemical compounds. The general Randic index of a graph $\Upsilon$ is defined as

2 $$\begin{aligned} GR(\Upsilon )&=\sum _{\upsilon ,\nu \in E(\Upsilon )}{(d(\upsilon )d(\nu ))}^{\gamma }. \end{aligned}$$

The Atom-Bond Connectivity (ABC) index^23^ is a mathematical tool in chemistry used to analyze the structure of molecules, measure their complexity. The mathematical formula of *ABC* index is

3 $$\begin{aligned} ABC(\Upsilon )&=\sum _{\upsilon ,\nu \in E(\Upsilon )}{\sqrt{\frac{d(\upsilon )+d(\nu )-2}{d(\upsilon )+d(\nu )}}}. \end{aligned}$$

It is used in Quantitative Structure-Activity Relationship studies, molecular descriptors and cheminformatics to study interactions, describe molecules and analyze chemical data^24,25^. The Atom-Bond Sum Connectivity (ABS) index^26^ is a topological index used in chemical graph theory to quantify the molecular structure of chemical compounds. It provides a numerical descriptor of molecular structure, useful in computational chemistry, quantitative structure-activity relationship studies, and other areas. It captures information about atom connectivity and bond types, enabling correlation with molecular properties and activities. It is defined as

4 $$\begin{aligned} ABS(\Upsilon )&=\sum _{\upsilon ,\nu \in E(\Upsilon )}{\sqrt{\frac{d(\upsilon )+d(\nu )-2}{d(\upsilon )d(\nu )}}}. \end{aligned}$$

The geometric arithmetic index^27^, which combines geometric and arithmetic mean values of molecular graph properties, helps chemists understand molecule structural characteristics and predict their behavior in chemical processes or biological activities. The geometric arithmetic index of a graph $\Upsilon$ has the mathematical formula

5 $$\begin{aligned} GA(\Upsilon )&=\sum _{\upsilon ,\nu \in E(\Upsilon )}\frac{2\sqrt{d(\upsilon )d(\nu )}}{d(\upsilon )+d(\nu )}. \end{aligned}$$

The paper is structured as follows: In Section “Materials and methods”, we discuss the 2D and 3D models of cyclodecanes and their properties. We explain the construction of random cyclodecane chains, and we have obtained general formulas for some connectivity-based topological indices. In Section “Main results and discussions”, we compute explicit expressions for the connectivity-based topological indices of random cyclodecane chains. The expressions for the expected values of these topological descriptors are obtained for some special cases. An analytical comparison between the expected values of these topological descriptors is presented in Section “Comparison between the expected values of topological descriptors”. Finally, the conclusion section summarizes the article.

## Materials and methods

**Figure 1:**
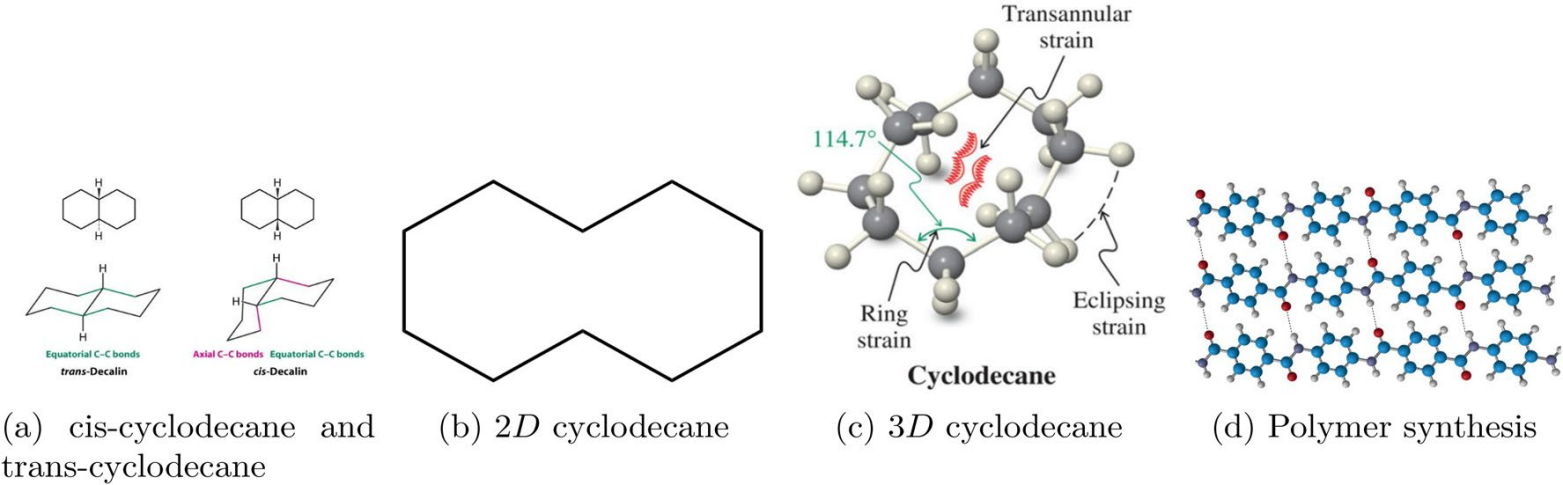
2D and 3D models of cyclodecanes.

Cyclodecane is a ten-carbon ring with ten membered rings, with two possible isomers, cis-cyclodecane and trans-cyclodecane (see Fig. 1). It undergoes Bergmann cyclization to produce diradical products that inhibit cell replication and interact with DNA. The 2D chemical structure of cyclodecane, also known as the skeletal formula, is the standard notation for organic molecules. Carbon atoms are located at the corner(s) and hydrogen atoms are not indicated. Each carbon atom is associated with enough hydrogen atoms to form four bonds. The 3D chemical structure image of cyclodecane uses a ball-and-stick model, displaying atom positions and bonds. The radius of spheres is smaller than rod lengths, allowing for a clearer view of atoms and bonds. In comparison to typical polymers, cyclodecane-based monomers enable polymer synthesis, resulting in unique polymers with cyclodecane-containing characteristics. Cyclodecane may impact the crystal structure of certain compounds, particularly those with coordination complexes or molecular assemblies, affecting the packing arrangement and overall properties of the crystal lattice. The chemical structure of a molecule contains the arrangement of its atoms and the bonds that hold them together. Cyclocodecane has 30 bonds, including 10 non-hydrogen bonds and 1 ten-numbered ring. The 2D and 3D models of cyclodecane chains are depicted in Fig. 1. The structure of the cyclodecane chain is chemical as well. Some of the characteristics of cyclodecane chains are: Molecular Weight 140.27 g/mol, Melting Point $10.0^{\circ }C$, Boiling Point $202.0^{\circ }C$, Health Risk 0.33 mg/L, Water Solubility $25^{\circ }C$ and Vapour Pressure 0.56 mmHg.

**Figure 2:**
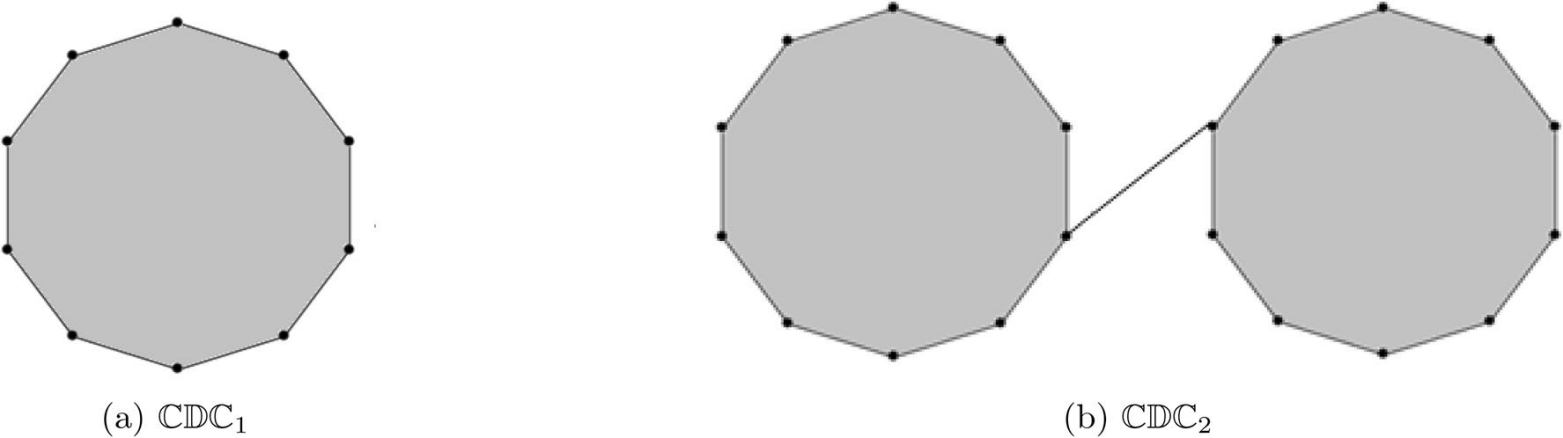
Cyclodecane chains for $k=1$ and $k=2$.

Researcher have focused on hydrocarbons and their derivatives because of their simple structure have two components carbon and hydrogen. Numerous kinds of hydrocarbon derivatives can be obtained by substituting their molecular hydrogen atoms with various other atomic groups. Plants contains a significant amount of precious hydrocarbons and some of these hydrocarbons properties are important in the production of chemical raw material and fuel. A cycloalkane with the chemical formula $C_{10}H_{20}$ is cyclodecane. When an edge is used to join the two or more decagons then it is known as cyclodecane chain. A random cyclodecane of length *k* is a chain containing *k* decagons which are connected to each other by edge in a random way. We use the notation $\mathbb {CDC}_{k}$ to denote a random cyclodecane chain containing *k* decagons. Figure 2 shows the unique cyclodecane $\mathbb {CDC}_{k}$ for $k=1,2$. There are five possible ways to connect a terminal decagon with the cyclodecane chain $\mathbb {CDC}_{k-1}$ with probability $\delta _{1}$, $\delta _{2}$, $\delta _{3}$, $\delta _{4}$, and $\delta _{5}=1-\delta _{1}-\delta _{2}-\delta _{3}-\delta _{4}$ respectively. A random selection is made from one of the five possibilities at each step $(q=3,4,5,...,k)$:

(i) $$\mathbb {CDC}_{q-1}\rightarrow \mathbb {CDC}^1_q$$ with probability $$\delta _{1}$$.
(ii) $\mathbb {CDC}_{q-1}\rightarrow \mathbb {CDC}^2_q$ with probability $\delta _{2}$.
(iii) $\mathbb {CDC}_{q-1}\rightarrow \mathbb {CDC}^3_q$ with probability $\delta _{3}$.
(iv) $\mathbb {CDC}_{q-1}\rightarrow \mathbb {CDC}^4_q$ with probability $\delta _{4}$.
(v) $$\mathbb {CDC}_{q-1}\rightarrow \mathbb {CDC}^5_q$$ with probability $$\delta _{5}=1-\delta _{1}-\delta _{2}-\delta _{3}-\delta _{4}$$.

**Figure 3:**
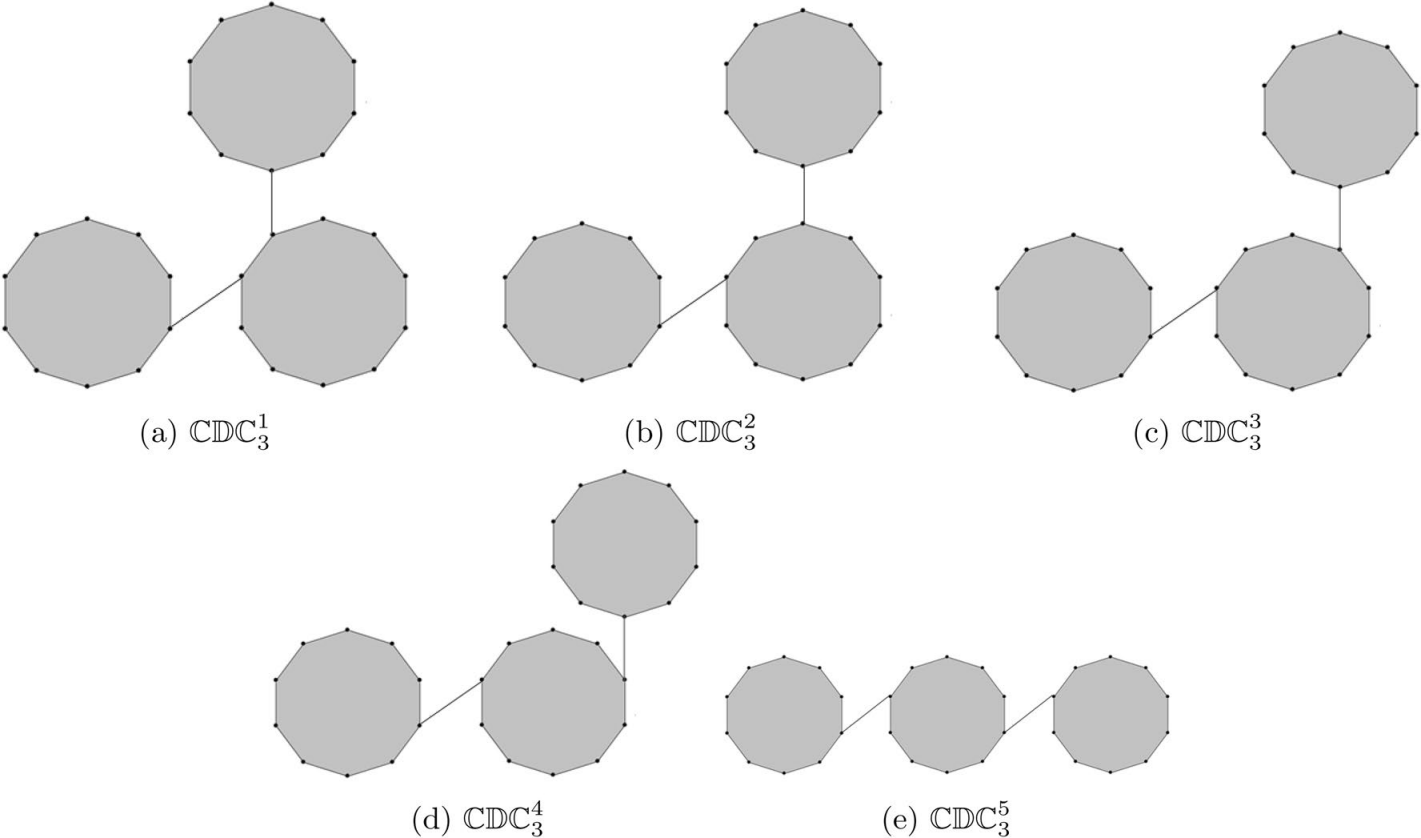
The five types of cyclodecane chain for $k=3.$

**Figure 4:**
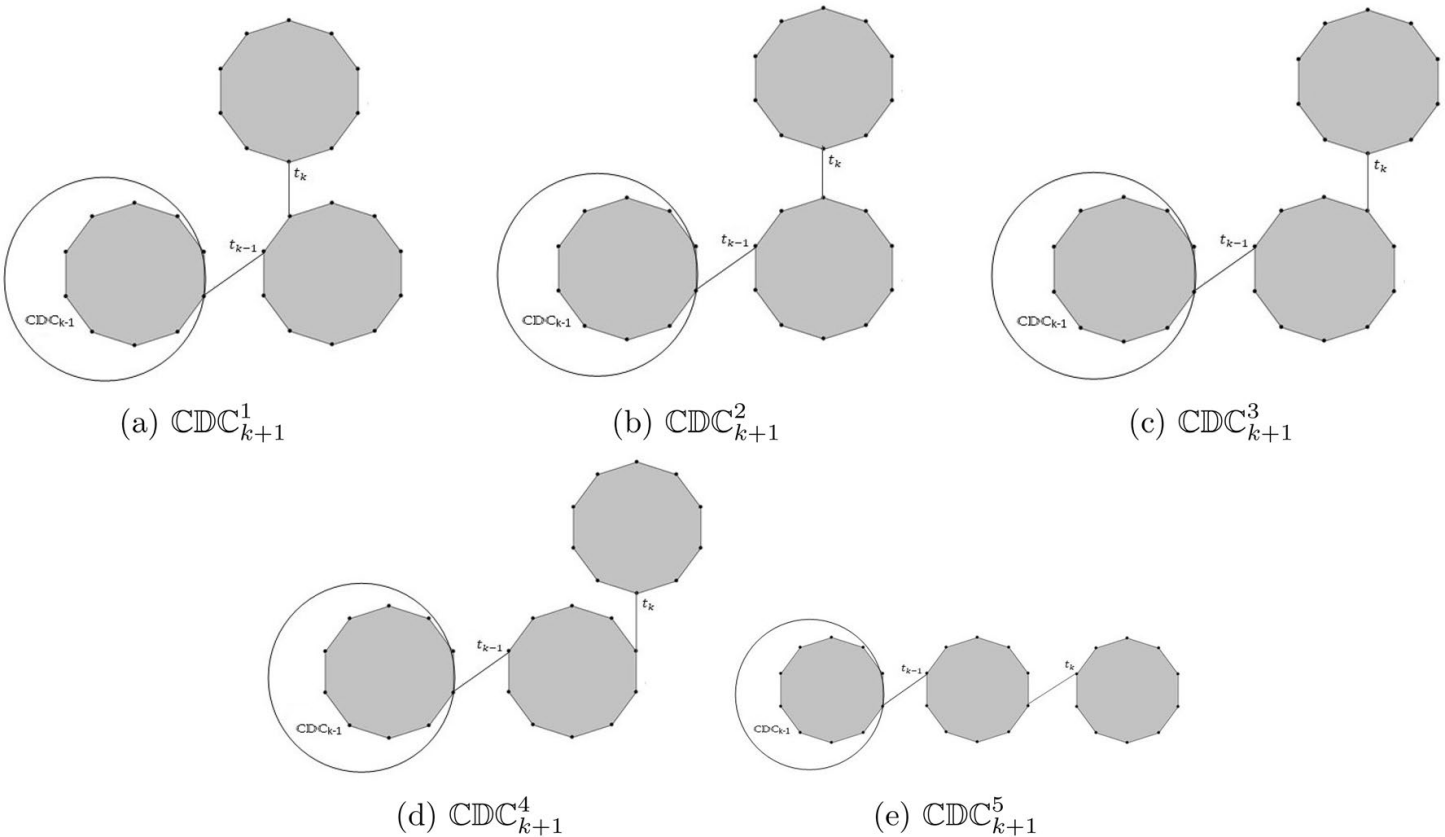
The five different configurations in cyclodecan for $k>3$.

For $k=3$, we have five different possible cyclodecane chains (see Fig. 3). The five different configurations of cyclodecane chains $\mathbb {CDC}^1_{k+1}$, $\mathbb {CDC}^2_{k+1}$, $\mathbb {CDC}^3_{k+1}$, $\mathbb {CDC}^4_{k+1}$ and $\mathbb {CDC}^5_{k+1}$ are shown in Fig. 4. For results on the expected values of different topological indices of random structures see^28–38^.

In this section, we compute the expected values of geometric-arithmetic index, atom-bound connectivity index, atom-bound-sum connectivity index, Randić index and general Randić index for $\mathbb {CDC}_{k}$ chain having *k* decagons. Consider $\mathbb {CDC}_{k}$ to be the cyclodecane chain formed from $\mathbb {CDC}_{k-1}$, as illustrated in Fig. 4. We use the notation $\upsilon _{ij}$ to denote the number of edges of $\mathbb {CDC}_{k}$ whose end vertices have degree *i* and *j* respectively. The structure of the chain $\mathbb {CDC}_{k}$ clearly shows that it comprises only (2, 2), (2, 3), and (3, 3) type edges. To calculate these indices for the chain $\mathbb {CDC}_{k}$, we need to find the edges of the type $\upsilon _{22}(\mathbb {CDC}_{k})$, $\upsilon _{23}(\mathbb {CDC}_{k})$ and $\upsilon _{33}(\mathbb {CDC}_{k})$. Using this information, Eqs. (1), (2), (3), (4) and (5) can be written as:

6 $$\begin{aligned} GA(\mathbb {CDC}_k)&=\upsilon _{22}(\mathbb {CDC}_{k}) + 0.9798 \upsilon _{23}(\mathbb {CDC}_{k}) + \upsilon _{33}(\mathbb {CDC}_{k}), \end{aligned}$$

7 $$\begin{aligned} ABC(\mathbb {CDC}_k)&=0.7071\upsilon _{22}(\mathbb {CDC}_{k}) + 0.7071 \upsilon _{23}(\mathbb {CDC}_{k}) + 0.6667 \upsilon _{33}(\mathbb {CDC}_{k}), \end{aligned}$$

8 $$\begin{aligned} ABS(\mathbb {CDC}_k)&=0.7071\upsilon _{22}(\mathbb {CDC}_{k}) + 0.7746 \upsilon _{23}(\mathbb {CDC}_{k}) + 0.8165\upsilon _{33}(\mathbb {CDC}_{k}), \end{aligned}$$

9 $$\begin{aligned} R(\mathbb {CDC}_k)&=0.5\upsilon _{22}(\mathbb {CDC}_{k}) + 0.4082 \upsilon _{23}(\mathbb {CDC}_{k}) + 0.3333 \upsilon _{33}(\mathbb {CDC}_{k}), \end{aligned}$$

10 $$\begin{aligned} GR(\mathbb {CDC}_k)&=4^{\gamma }\upsilon _{22}(\mathbb {CDC}_{k}) + 6^{\gamma } \upsilon _{23}(\mathbb {CDC}_{k}) +9^{\gamma } \upsilon _{33}(\mathbb {CDC}_{k}). \end{aligned}$$

## Main results and discussions

For $k\ge 3$, the cyclodecane chain $\mathbb {CDC}_{k}$ is a random structure. It follows $GA(\mathbb {CDC}_k)$, $ABC(\mathbb {CDC}_k)$, $ABS(\mathbb {CDC}_k)$, $R(\mathbb {CDC}_k)$ and $GR(\mathbb {CDC}_k)$ are random variables. We use the notaions $E^{GA}(\mathbb {CDC}_k)=E[GA(\mathbb {CDC}_k)]$, $E^{ABC}(\mathbb {CDC}_k)=E[ABC(\mathbb {CDC}_k)]$, $E^{ABS}(\mathbb {CDC}_k)=E[ABS(\mathbb {CDC}_k)]$, $E^{R}(\mathbb {CDC}_k)=E[R(\mathbb {CDC}_k)]$ and $E^{GR}(\mathbb {CDC}_k)=E[GR(\mathbb {CDC}_k)]$ to denote their expected values respectively.

### Theorem 1

Let $k\ge 2$, then the expected value of the Geometric-Arithmetic index of $\mathbb {CDC}_k$ is

$$\begin{aligned} E^{GA}(\mathbb {CDC}_k)= k(0.0404\delta _{1}+10.9192)-0.0808\delta _{1}-0.9192. \end{aligned}$$

### Proof

For $k=2$, we get $E^{GA}(\mathbb {CDC}_2) = 20.9192$ which is indeed true. Let $k \ge 3$, then there are five possibilities.

a) If $\mathbb {CDP}_{-k1} \longrightarrow \mathbb {CDC}^{1}_{k}$, then $\upsilon _{22}(\mathbb {CDC}^{1}_{k}) = \upsilon _{22}(\mathbb {CDC}_{k-1})+7$, $\upsilon _{23}(\mathbb {CDC}^{1}_{k}) = \upsilon _{23}(\mathbb {CDC}_{k-1})+2$ and $\upsilon _{33}(\mathbb {CDC}^{1}_{k}) = \upsilon _{33}(\mathbb {CDC}_{k-1})+2$. Using these values in Eq. (6), we get

$$\begin{aligned} GA(\mathbb {CDC}^{1}_{k}) = GA(\mathbb {CDC}_{k-1})+ 10.9596. \end{aligned}$$

b) If $\mathbb {CDC}_{k-1} \longrightarrow \mathbb {CDC}^{2}_{k}$, then $\upsilon _{22}(\mathbb {CDC}^{2}_{k}) = \upsilon _{22}(\mathbb {CDC}_{k-1})+6$, $\upsilon _{23}(\mathbb {CDC}^{2}_{k}) = \upsilon _{23}(\mathbb {CDC}_{k-1})+4$ and $\upsilon _{33}(\mathbb {CDC}^{2}_{k}) = \upsilon _{33}(\mathbb {CDC}_{k-1})+1$. Using these values in Eq. (6), we get

$$\begin{aligned} GA(\mathbb {CDC}^{2}_{k}) = GA(\mathbb {CDC}_{k-1})+ 10.9192. \end{aligned}$$

c) If $\mathbb {CDC}_{k-1} \longrightarrow \mathbb {CDC}^{3}_{k}$, then $\upsilon _{22}(\mathbb {CDC}^{3}_{k}) = \upsilon _{22}(\mathbb {CDC}_{k-1})+6$, $\upsilon _{23}(\mathbb {CDC}^{3}_{k}) = \upsilon _{23}(\mathbb {CDC}_{k-1})+4$ and $\upsilon _{33}(\mathbb {CDC}^{3}_{k}) = \upsilon _{33}(\mathbb {CDC}_{k-1})+1$. Using these values in Eq. (6), we get

$$\begin{aligned} GA(\mathbb {CDC}^{3}_{k}) = GA(\mathbb {CDC}_{k-1})+ 10.9192. \end{aligned}$$

d)If $\mathbb {CDC}_{k-1} \longrightarrow \mathbb {CDC}^{4}_{k}$, then $\upsilon _{22}(\mathbb {CDC}^{4}_{k}) = \upsilon _{22}(\mathbb {CDC}_{k-1})+6$, $\upsilon _{23}(\mathbb {CDC}^{4}_{k}) = \upsilon _{23}(\mathbb {CDC}_{k-1})+4$ and $\upsilon _{33}(\mathbb {CDC}^{4}_{k}) = \upsilon _{33}(\mathbb {CDC}_{k-1})+1$. Using these values in Eq. (6), we get

$$\begin{aligned} GA(\mathbb {CDC}^{4}_{k}) = GA(\mathbb {CDC}_{k-1})+ 10.9192. \end{aligned}$$

e) If $\mathbb {CDC}_{k-1} \longrightarrow \mathbb {CDC}^{5}_{k}$, then $\upsilon _{22}(\mathbb {CDC}^{5}_{k}) = \upsilon _{22}(\mathbb {CDC}_{k-1})+6$, $\upsilon _{23}(\mathbb {CDC}^{5}_{k}) = \upsilon _{23}(\mathbb {CDC}_{k-1})+4$ and $\upsilon _{33}(\mathbb {CDC}^{5}_{k}) = \upsilon _{33}(\mathbb {CDC}_{k-1})+1$. Using these values in Eq. (6), we get

$$\begin{aligned} GA(\mathbb {CDC}^{5}_{k}) = GA(\mathbb {CDC}_{k-1})+ 10.9192. \end{aligned}$$

Thus, we have

$$\begin{aligned} E^{GA}(\mathbb {CDC}_k)= & {} \delta _{1} GA(\mathbb {CDC}^{1}_{k})+ \delta _{2} GA(\mathbb {CDC}^{2}_{k})+ \delta _{3} GA(\mathbb {CDC}^{3}_{k})+\delta _{4}GA(\mathbb {CDC}^{4}_{k})\\{} & {} \displaystyle +(1-\delta _{1}-\delta _{2}-\delta _{3}-\delta _{4}) GA(\mathbb {CDC}^{5}_{k})\\= & {} GA(\mathbb {CDC}_{k-1}) + 0.0404\delta _{1}+ 10.9192. \end{aligned}$$

Since $E[E^{GA}(\mathbb {CDC}_k)] = E^{GA}(\mathbb {CDC}_k)$, it follows that

$$\begin{aligned} E^{GA}(\mathbb {CDC}_k)&= E^{GA}(\mathbb {CDC}_{k-1}) + 0.0404\delta _{1}+10.9192. \end{aligned}$$

Finally, solving the the recurrence relation by using the initial condition $E(\mathbb {CDC}_2)=20.9192$, we get

$$\begin{aligned} E^{GA}(\mathbb {CDC}_k)&= k(0.0404\delta _{1}+10.9192)-0.0808\delta _{1}-0.9192. \end{aligned}$$

$$\square $$

### Theorem 2

Let $k\ge 2$, then the expected value of the atom-bound connectivity index of $\mathbb {CDC}_k$ is

$$\begin{aligned} E^{ABC}(\mathbb {CDC}_k)= k(7.7377-0.0404\delta _{1})+0.0809\delta _{1}-7.0711. \end{aligned}$$

### Proof

For $k=2$, we get $E^{ABC}(\mathbb {CDC}_2) = 14.81$ which is indeed true. Let $k \ge 3$, then there are five possibilities.

a) If $\mathbb {CDC}_{k-1} \longrightarrow \mathbb {CDC}^{1}_{k}$, then $\upsilon _{22}(\mathbb {CDC}^{1}_{k}) = \upsilon _{22}(\mathbb {CDC}_{k-1})+7$, $\upsilon _{23}(\mathbb {CDC}^{1}_{k}) = \upsilon _{23}(\mathbb {CDC}_{k-1})+2$ and $\upsilon _{33}(\mathbb {CDC}^{1}_{k}) = \upsilon _{33}(\mathbb {CDC}_{k-1})+2$. Using these values in Eq. (7), we get

$$\begin{aligned} ABC(\mathbb {CDC}^{1}_{k}) = ABC(\mathbb {CDC}_{k-1})+ 7.6973. \end{aligned}$$

b) If $\mathbb {CDC}_{k-1} \longrightarrow \mathbb {CDC}^{2}_{k}$, then $\upsilon _{22}(\mathbb {CDC}^{2}_{k}) = \upsilon _{22}(\mathbb {CDC}_{k-1})+6$, $\upsilon _{23}(\mathbb {CDC}^{2}_{k}) = \upsilon _{23}(\mathbb {CDC}_{k-1})+4$ and $\upsilon _{33}(\mathbb {CDC}^{2}_{k}) = \upsilon _{33}(\mathbb {CDC}_{k-1})+1$. Using these values in Eq. (7), we get

$$\begin{aligned} ABC(\mathbb {CDC}^{2}_{k}) = ABC(\mathbb {CDC}_{k-1})+ 7.7377. \end{aligned}$$

c) If $\mathbb {CDC}_{k-1} \longrightarrow \mathbb {CDC}^{3}_{k}$, then $\upsilon _{22}(\mathbb {CDC}^{3}_{k}) = \upsilon _{22}(\mathbb {CDC}_{k-1})+6$, $\upsilon _{23}(\mathbb {CDC}^{3}_{k}) = \upsilon _{23}(\mathbb {CDC}_{k-1})+4$ and $\upsilon _{33}(\mathbb {CDC}^{3}_{k}) = \upsilon _{33}(\mathbb {CDC}_{k-1})+1$. Using these values in Eq. (7), we get

$$\begin{aligned} ABC(\mathbb {CDC}^{3}_{k}) = ABC(\mathbb {CDC}_{k-1})+ 7,7377. \end{aligned}$$

d)If $\mathbb {CDC}_{k-1} \longrightarrow \mathbb {CDC}^{4}_{k}$, then $\upsilon _{22}(\mathbb {CDC}^{4}_{k}) = \upsilon _{22}(\mathbb {CDC}_{k-1})+6$, $\upsilon _{23}(\mathbb {CDC}^{4}_{k}) = \upsilon _{23}(\mathbb {CDC}_{k-1})+4$ and $\upsilon _{33}(\mathbb {CDC}^{4}_{k}) = \upsilon _{33}(\mathbb {CDC}_{k-1})+1$. Using these values in Eq. (7), we get

$$\begin{aligned} ABC(\mathbb {CDC}^{4}_{k}) = ABC(\mathbb {CDC}_{k-1})+ 7.7377. \end{aligned}$$

e)If $\mathbb {CDC}_{k-1} \longrightarrow \mathbb {CDC}^{5}_{k}$, then $\upsilon _{22}(\mathbb {CDC}^{5}_{k}) = \upsilon _{22}(\mathbb {CDC}_{k-1})+6$, $\upsilon _{23}(\mathbb {CDC}^{5}_{k}) = \upsilon _{23}(\mathbb {CDC}_{k-1})+4$ and $\upsilon _{33}(\mathbb {CDC}^{5}_{k}) = \upsilon _{33}(\mathbb {CDC}_{k-1})+1$. Using these values in Eq. (7), we get

$$\begin{aligned} ABC(\mathbb {CDC}^{5}_{k}) = ABC(\mathbb {CDC}_{k-1})+ 7.7377. \end{aligned}$$

Thus, we have

$$\begin{aligned} E^{ABC}(\mathbb {CDC}_k)= & {} \delta _{1} ABC(\mathbb {CDC}^{1}_{k})+ \delta _{2} ABC(\mathbb {CDC}^{2}_{k})+ \delta _{3} ABC(\mathbb {CDC}^{3}_{k})+ \delta _{4} ABC(\mathbb {CDC}^{4}_{k})\\{} & {} \displaystyle +(1-\delta _{1}-\delta _{2}-\delta _{3}-\delta _{4}) ABC(\mathbb {CDC}^{5}_{k}).\\= & {} ABC(\mathbb {CDC}_{k-1}) -0.0404\delta _{1}+ 7.7377. \end{aligned}$$

since $E[E^{ABC}(\mathbb {CDC}_k) ] = E^{ABC}(\mathbb {CDC}_k)$, it follows that

$$\begin{aligned} E^{ABC}(\mathbb {CDC}_k)&= E^{ABC}(\mathbb {CDC}_{k-1})-0.0404\delta _{1}+ 7.7377. \end{aligned}$$

Finally, solving the the recurrence relation by using the initial condition $E(\mathbb {CDC}_2)=14.81$, we get

$$\begin{aligned} E^{ABC}(\mathbb {CDC}_k)&= k(7.7377-0.0404\delta _{1})+0.0809\delta _{1}-7.0711. \end{aligned}$$

$$\square $$

### Theorem 3

Let $k\ge 2$, then the expected value of the atom-bound-sum connectivity index of $\mathbb {CDC}_k$ is

$$\begin{aligned} E^{ABS}(\mathbb {CDC}_k)= k(8.1575-0.0256\delta _{1})+0.0512\delta _{1}-1.0864. \end{aligned}$$

### Proof

For $k=2$, we get $E^{ABS}(\mathbb {CDC}_2) = 15.2286$ which is indeed true. Let $k \ge 3$, then there are five possibilities.

a) If $\mathbb {CDC}_{k-1} \longrightarrow \mathbb {CDC}^{1}_{k}$, then $\upsilon _{22}(\mathbb {CDC}^{1}_{k}) = \upsilon _{22}(\mathbb {CDC}_{k-1})+7$, $\upsilon _{23}(\mathbb {CDC}^{1}_{k}) = \upsilon _{23}(\mathbb {CDC}_{k-1})+2$ and $\upsilon _{33}(\mathbb {CDC}^{1}_{k}) = \upsilon _{33}(\mathbb {CDC}_{k-1})+2$. Using these values in Eq. (8), we get

$$\begin{aligned} ABS(\mathbb {CDC}^{1}_{k}) = ABS(\mathbb {CDC}_{k-1})+ 8.1319. \end{aligned}$$

b) If $\mathbb {CDC}_{k-1} \longrightarrow \mathbb {CDC}^{2}_{k}$, then $\upsilon _{22}(\mathbb {CDC}^{2}_{k}) = \upsilon _{22}(\mathbb {CDC}_{k-1})+6$, $\upsilon _{23}(\mathbb {CDC}^{2}_{k}) = \upsilon _{23}(\mathbb {CDC}_{k-1})+4$ and $\upsilon _{33}(\mathbb {CDC}^{2}_{k}) = \upsilon _{33}(\mathbb {CDC}_{k-1})+1$. Using these values in Eq. (8), we get

$$\begin{aligned} ABS(\mathbb {CDC}^{2}_{k}) = ABS(\mathbb {CDC}_{k-1})+ 8.1575. \end{aligned}$$

c) If $\mathbb {CDC}_{k-1} \longrightarrow \mathbb {CDC}^{3}_{k}$, then $\upsilon _{22}(\mathbb {CDC}^{3}_{k}) = \upsilon _{22}(\mathbb {CDC}_{k-1})+6$, $\upsilon _{23}(\mathbb {CDC}^{3}_{k}) = \upsilon _{23}(\mathbb {CDC}_{k-1})+4$ and $\upsilon _{33}(\mathbb {CDC}^{3}_{k}) = \upsilon _{33}(\mathbb {CDC}_{k-1})+1$. Using these values in Eq. (8), we get

$$\begin{aligned} ABS(\mathbb {CDC}^{3}_{k}) = ABS(\mathbb {CDC}_{k-1})+ 8.1575. \end{aligned}$$

d)If $\mathbb {CDC}_{k-1} \longrightarrow \mathbb {CDC}^{4}_{k}$, then $\upsilon _{22}(\mathbb {CDC}^{4}_{k}) = \upsilon _{22}(\mathbb {CDC}_{k-1})+6$, $\upsilon _{23}(\mathbb {CDC}^{4}_{k}) = \upsilon _{23}(\mathbb {CDC}_{k-1})+4$ and $\upsilon _{33}(\mathbb {CDC}^{4}_{k}) = \upsilon _{33}(\mathbb {CDC}_{k-1})+1$. Using these values in Eq. (8), we get

$$\begin{aligned} ABS(\mathbb {CDC}^{4}_{k}) = ABS(\mathbb {CDC}_{k-1})+ 8.1575. \end{aligned}$$

e)If $\mathbb {CDC}_{k-1} \longrightarrow \mathbb {CDC}^{5}_{k}$, then $\upsilon _{22}(\mathbb {CDC}^{5}_{k}) = \upsilon _{22}(\mathbb {CDC}_{k-1})+6$, $\upsilon _{23}(\mathbb {CDC}^{5}_{k}) = \upsilon _{23}(\mathbb {CDC}_{k-1})+4$ and $\upsilon _{33}(\mathbb {CDC}^{5}_{k}) = \upsilon _{33}(\mathbb {CDC}_{k-1})+1$. Using these values in Eq. (8), we get

$$\begin{aligned} ABS(\mathbb {CDC}^{5}_{k}) = ABS(\mathbb {CDC}_{k-1})+ 8.1575. \end{aligned}$$

Thus, we have

$$\begin{aligned} E^{ABS}(\mathbb {CDC}_k)&= \delta _{1} ABS(\mathbb {CDC}^{1}_{k})+ \delta _{2} ABS(\mathbb {CDC}^{2}_{k})+ \delta _{3} ABS(\mathbb {CDC}^{3}_{k})\\&\quad \displaystyle + \delta _{4} ABS(\mathbb {CDC}^{4}_{k})+(1-\delta _{1}-\delta _{2}-\delta _{3}-\delta _{4}) ABS(\mathbb {CDC}^{5}_{k}).\\&=ABS(\mathbb {CDC}_{k-1}) -0.0256\delta _{1}+ 8.1575. \end{aligned}$$

Since $E[E^{ABS}(\mathbb {CDC}_k) ] = E^{ABS}(\mathbb {CDC}_k)$, it follows that

$$\begin{aligned} E^{ABS}(\mathbb {CDC}_k)&= E^{ABS}(\mathbb {CDC}_{k-1})-0.0256\delta _{1}+ 8.1575. \end{aligned}$$

Finally, solving the the recurrence relation by using the initial condition $E(\mathbb {CDC}_2)=15.2286$, we get

$$\begin{aligned} E^{ABS}(\mathbb {CDC}_k)&= (k)(8.1575-0.0256\delta _{1})+0.0512\delta _{1}-1.0864. \end{aligned}$$

$$\square $$

### Theorem 4

Let $k\ge 2$, then the expected value of the Randić index of $\mathbb {CDC}_k$ is

$$\begin{aligned} E^{R}(\mathbb {CDC}_k)= k(0.0169\delta _{1}+4.9663)-0.0338\delta _{1}+0.0337. \end{aligned}$$

### Proof

For $k=2$, we get $E^{R}(\mathbb {CDC}_2) = 9.9663$ which is indeed true. Let $k \ge 3$, then there are five possibilities.

a) If $\mathbb {CDC}_{k-1} \longrightarrow \mathbb {CDC}^{1}_{k}$, then $\upsilon _{22}(\mathbb {CDC}^{1}_{k}) = \upsilon _{22}(\mathbb {CDC}_{k-1})+7$, $\upsilon _{23}(\mathbb {CDC}^{1}_{k}) = \upsilon _{23}(\mathbb {CDC}_{k-1})+2$ and $\upsilon _{33}(\mathbb {CDC}^{1}_{k}) = \upsilon _{33}(\mathbb {CDC}_{k-1})+2$. Using these values in Eq. (9), we get

$$\begin{aligned} R(\mathbb {CDC}^{1}_{k}) = R(\mathbb {CDC}_{k-1})+ 4.9832. \end{aligned}$$

b) If $\mathbb {CDC}_{k-1} \longrightarrow \mathbb {CDC}^{2}_{k}$, then $\upsilon _{22}(\mathbb {CDC}^{2}_{k}) = \upsilon _{22}(\mathbb {CDC}_{k-1})+6$, $\upsilon _{23}(\mathbb {CDC}^{2}_{k}) = \upsilon _{23}(\mathbb {CDC}_{k-1})+4$ and $\upsilon _{33}(\mathbb {CDC}^{2}_{k}) = \upsilon _{33}(\mathbb {CDC}_{k-1})+1$. Using these values in Eq. (9), we get

$$\begin{aligned} R(\mathbb {CDC}^{2}_{k}) = R(\mathbb {CDC}_{k-1})+ 4.9663. \end{aligned}$$

c) If $\mathbb {CDC}_{k-1} \longrightarrow \mathbb {CDC}^{3}_{k}$, then $\upsilon _{22}(\mathbb {CDC}^{3}_{k}) = \upsilon _{22}(\mathbb {CDC}_{k-1})+6$, $\upsilon _{23}(\mathbb {CDC}^{3}_{k}) = \upsilon _{23}(\mathbb {CDC}_{k-1})+4$ and $\upsilon _{33}(\mathbb {CDC}^{3}_{k}) = \upsilon _{33}(\mathbb {CDC}_{k-1})+1$. Using these values in Eq. (9), we get

$$\begin{aligned} R(\mathbb {CDC}^{3}_{k}) = R(\mathbb {CDC}_{k-1})+ 4.9663. \end{aligned}$$

d)If $\mathbb {CDC}_{k-1} \longrightarrow \mathbb {CDC}^{4}_{k}$, then $\upsilon _{22}(\mathbb {CDC}^{4}_{k}) = \upsilon _{22}(\mathbb {CDC}_{k-1})+6$, $\upsilon _{23}(\mathbb {CDC}^{4}_{k}) = \upsilon _{23}(\mathbb {CDC}_{k-1})+4$ and $\upsilon _{33}(\mathbb {CDC}^{4}_{k}) = \upsilon _{33}(\mathbb {CDC}_{k-1})+1$. Using these values in Eq. (9), we get

$$\begin{aligned} R(\mathbb {CDC}^{4}_{k}) = R(\mathbb {CDC}_{k-1})+ 4,9663. \end{aligned}$$

e) If $\mathbb {CDC}_{k-1} \longrightarrow \mathbb {CDC}^{5}_{k}$, then $\upsilon _{22}(\mathbb {CDC}^{5}_{k}) = \upsilon _{22}(\mathbb {CDC}_{k-1})+6$, $\upsilon _{23}(\mathbb {CDC}^{5}_{k}) = \upsilon _{23}(\mathbb {CDC}_{k-1})+4$ and $\upsilon _{33}(\mathbb {CDC}^{5}_{k}) = \upsilon _{33}(\mathbb {CDC}_{k-1})+1$. Using these values in Eq. (9), we get

$$\begin{aligned} R(\mathbb {CDC}^{5}_{k}) = R(\mathbb {CDC}_{k-1})+ 4,9663. \end{aligned}$$

Thus, we have

$$\begin{aligned} E^{R}(\mathbb {CDC}_k)= & {} \delta _{1} R(\mathbb {CDC}^{1}_{k})+ \delta _{2} R(\mathbb {CDC}^{2}_{k})+ \delta _{3}R(\mathbb {CDC}^{3}_{k})+\delta _{4}R(\mathbb {CDC}^{4}_{k})\\{} & {} \displaystyle +(1-\delta _{1}-\delta _{2}-\delta _{3}-\delta _{4}) R(\mathbb {CDC}^{5}_{k})\\= & {} R(\mathbb {CDC}_{k-1}) + 0.0169\delta _{1}+ 4.9663. \end{aligned}$$

Since $E[E^{R}(\mathbb {CDC}_k) ] = E^{R}(\mathbb {CDC}_k)$, it follows that

$$\begin{aligned} E^{R}(\mathbb {CDC}_k)&= E^{R}(\mathbb {CDC}_{k-1}) + 0.0169\delta _{1}+ 4.9663. \end{aligned}$$

Finally, solving the the recurrence relation by using the initial condition $E(\mathbb {CDC}_2)=9.9663$, we get

$$\begin{aligned} E^{R}(\mathbb {CDC}_k)&= k(0.0169\delta _{1}+4.9663)-0.0338\delta _{1}+0.0337. \end{aligned}$$

$$\square $$

### Theorem 5

Let $k\ge 2$, then the expected value of the general Randić index of $\mathbb {CDC}_k$ is

$$\begin{aligned} E^{GR}(\mathbb {CDC}_k)= k[(4^{\gamma }-2(6^{\gamma })+9^{\gamma })\delta _{1}+6(4^{\gamma })+4(6^{\gamma })+9^{\gamma }] -2(4^{\gamma }-2(6^{\gamma })+9^{\gamma })\delta _{1}+4(4^{\gamma })+4(6^{\gamma })-9^{\gamma }. \end{aligned}$$

### Proof

For $k=2$, we get $E^{R}(\mathbb {CDC}_2) = 16(4^{\gamma })+4(6^{\gamma })+9^{\gamma }$ which is indeed true. Let $k \ge 3$, then there are five possibilities.

a) If $\mathbb {CDC}_{k-1} \longrightarrow \mathbb {CDC}^{1}_{k}$, then $\upsilon _{22}(\mathbb {CDC}^{1}_{k}) = \upsilon _{22}(\mathbb {CDC}_{k-1})+7$, $\upsilon _{23}(\mathbb {CDC}^{1}_{k}) = \upsilon _{23}(\mathbb {CDC}_{k-1})+2$ and $\upsilon _{33}(\mathbb {CDC}^{1}_{k}) = \upsilon _{33}(\mathbb {CDC}_{k-1})+2$. Using these values in Eq. (10), we get

$$\begin{aligned} GR(\mathbb {CDC}^{1}_{k}) = GR(\mathbb {CDC}_{k-1})+ 7(4^{\gamma })+2(6^{\gamma })+2(9^{\gamma }). \end{aligned}$$

b) If $\mathbb {CDC}_{k-1} \longrightarrow \mathbb {CDC}^{2}_{k}$, then $\upsilon _{22}(\mathbb {CDC}^{2}_{k}) = \upsilon _{22}(\mathbb {CDC}_{k-1})+6$, $\upsilon _{23}(\mathbb {CDC}^{2}_{k}) = \upsilon _{23}(\mathbb {CDC}_{k-1})+4$ and $\upsilon _{33}(\mathbb {CDC}^{2}_{k}) = \upsilon _{33}(\mathbb {CDC}_{k-1})+1$. Using these values in Eq. (10), we get

$$\begin{aligned} GR(\mathbb {CDC}^{2}_{k}) = GR(\mathbb {CDC}_{k-1})+ 6(4^{\gamma })+4(6^{\gamma })+9^{\gamma }. \end{aligned}$$

c) If $\mathbb {CDC}_{k-1} \longrightarrow \mathbb {CDC}^{3}_{k}$, then $\upsilon _{22}(\mathbb {CDC}^{3}_{k}) = \upsilon _{22}(\mathbb {CDC}_{k-1})+6$, $\upsilon _{23}(\mathbb {CDC}^{3}_{k}) = \upsilon _{23}(\mathbb {CDC}_{k-1})+4$ and $\upsilon _{33}(\mathbb {CDC}^{3}_{k}) = \upsilon _{33}(\mathbb {CDC}_{k-1})+1$. Using these values in Eq. (10), we get

$$\begin{aligned} GR(\mathbb {CDC}^{3}_{k}) = GR(\mathbb {CDC}_{k-1})+6(4^{\gamma })+4(6^{\gamma })+9^{\gamma }. \end{aligned}$$

d)If $\mathbb {CDC}_{k-1} \longrightarrow \mathbb {CDC}^{4}_{k}$, then $\upsilon _{22}(\mathbb {CDC}^{4}_{k}) = \upsilon _{22}(\mathbb {CDC}_{k-1})+6$, $\upsilon _{23}(\mathbb {CDC}^{4}_{k}) = \upsilon _{23}(\mathbb {CDC}_{k-1})+4$ and $\upsilon _{33}(\mathbb {CDC}^{4}_{k}) = \upsilon _{33}(\mathbb {CDC}_{k-1})+1$. Using these values in Eq. (10), we get

$$\begin{aligned} GR(\mathbb {CDC}^{4}_{k}) = GR(\mathbb {CDC}_{k-1})+ 6(4^{\gamma })+4(6^{\gamma })+9^{\gamma }. \end{aligned}$$

e) If $\mathbb {CDC}_{k-1} \longrightarrow \mathbb {CDC}^{5}_{k}$, then $\upsilon _{22}(\mathbb {CDC}^{5}_{k}) = \upsilon _{22}(\mathbb {CDC}_{k-1})+6$, $\upsilon _{23}(\mathbb {CDC}^{5}_{k}) = \upsilon _{23}(\mathbb {CDC}_{k-1})+4$ and $\upsilon _{33}(\mathbb {CDC}^{5}_{k}) = \upsilon _{33}(\mathbb {CDC}_{k-1})+1$. Using these values in Eq. (10), we get

$$\begin{aligned} GR(\mathbb {CDC}^{5}_{k}) = GR(\mathbb {CDC}_{k-1})+ 6(4^{\gamma })+4(6^{\gamma })+9^{\gamma }. \end{aligned}$$

Thus, we have

$$\begin{aligned} E^{GR}(\mathbb {CDC}_k)= & {} \delta _{1} GR(\mathbb {CDC}^{1}_{k})+ \delta _{2} GR(\mathbb {CDC}^{2}_{k})+ \delta _{3}GR(\mathbb {CDC}^{3}_{k})+\delta _{4}GR(\mathbb {CDC}^{4}_{k})\\{} & {} \displaystyle +(1-\delta _{1}-\delta _{2}-\delta _{3}-\delta _{4})GR(\mathbb {CDC}^{5}_{k})\\= & {} GR(\mathbb {CDC}_{k-1}) + [(4^{\gamma })-2(6^{\gamma })+9^{\gamma }]\delta _{1}+ 6(4^{\gamma })+4(6^{\gamma })+9^{\gamma } \end{aligned}$$

Since $E[E^{GR}(\mathbb {CDC}_k) ] = E^{GR}(\mathbb {CDC}_k)$, it follows that

$$\begin{aligned} E^{GR}(\mathbb {CDC}_k)&= E^{GR}(\mathbb {CDC}_{k-1})+ [4^{\gamma }-2(6^{\gamma })+9^{\gamma }]\delta _{1}+ 6(4^{\gamma })+4(6^{\gamma })+9^{\gamma }. \end{aligned}$$

Finally, solving the the recurrence relation by using the initial condition $E(\mathbb {CDC}_2)= 7(4^{\gamma })+2(6^{\gamma })+2(9^{\gamma })$, we get

$$\begin{aligned} E^{GR}(\mathbb {CDC}_k)= & {} k[(4^{\gamma }-2(6^{\gamma })+9^{\gamma })\delta _{1}+6(4^{\gamma })+4(6^{\gamma })+9^{\gamma }]\\{} & {} \displaystyle -2(4^{\gamma }-2(6^{\gamma })+9^{\gamma })\delta _{1}+4(4^{\gamma })+4(6^{\gamma })-9^{\gamma }.\\ \end{aligned}$$

$$\square $$

**Figure 5:**
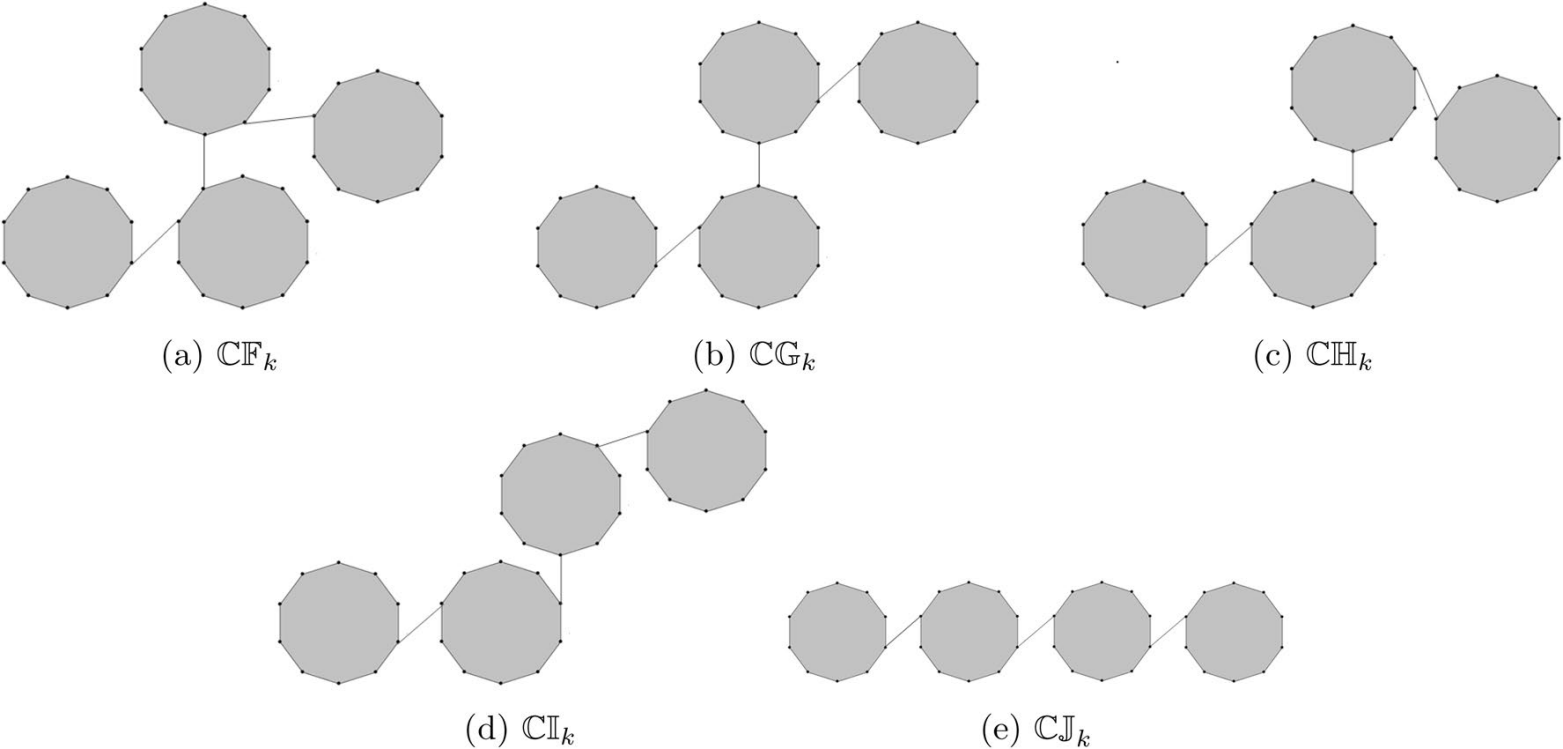
Five special cyclodecane chains with *k* decanes.

We now focus on the unique cyclodecane chains $\mathbb{C}\mathbb{F}_k$, $\mathbb{C}\mathbb{G}_k$, $\mathbb{C}\mathbb{H}_k$, $\mathbb{C}\mathbb{I}_k$ and $\mathbb{C}\mathbb{J}_k$ (see Fig. 5). These chains can be obtained from $\mathbb {CDC}_k$ as special cases by taking the value of one of the probability $\delta _i=1$ and the remaining probabilities 0 at each step, where $i=1,2,\ldots , 5$. We use Theorems 1, 2, 3 and 4 to calculate the topological indices for these five specific chains.

### Corollary 6

Let $k\ge 2$, then we have

- $$E^{ABC}(\mathbb{C}\mathbb{F}_k)=7.6973k-6.990$$.
- $$E^{ABS}(\mathbb{C}\mathbb{F}_k)=8.1319k-1.0352$$.
- $$E^{GA}(\mathbb{C}\mathbb{F}_k)=10.9596k-1$$.
- $$E^{R}(\mathbb{C}\mathbb{F}_k)=4.9832k-0.0001$$.
- $$E^{ABC}(\mathbb{C}\mathbb{G}_k)=ABC(\mathbb{C}\mathbb{H}_k)=ABC(\mathbb{C}\mathbb{I}_k)=ABC(\mathbb{C}\mathbb{J}_k)=7.7377k-7.0711$$.
- $$E^{ABS}(\mathbb{C}\mathbb{G}_k)=ABS(\mathbb{C}\mathbb{H}_k)=ABS(\mathbb{C}\mathbb{I}_k)=ABS(\mathbb{C}\mathbb{J}_k)=8.1575k-1.0864$$.
- $$E^{GA}(\mathbb{C}\mathbb{G}_k)=GA(\mathbb{C}\mathbb{H}_k)=GA(\mathbb{C}\mathbb{I}_k)=GA(\mathbb{C}\mathbb{J}_k)=10.9192k-0.9192$$.
- $$E^{R}(\mathbb{C}\mathbb{G}_k)=R(\mathbb{C}\mathbb{H}_k)=R(\mathbb{C}\mathbb{I}_k)=R(\mathbb{C}\mathbb{J}_k)=4.9663k+0.0337$$.

**Table 1:** The expected values of topological indices for $\delta _{1}=1$.

*k*	$E^{GA}$	$E^{ABC}$	$E^{ABS}$	$E^{R}$
4	42.8384	23.6799	31.4924	19.9327
5	53.798	31.4963	39.6242	24.9159
6	64.7576	39.1936	47.7562	29.8991
7	75.7172	46.8909	55.8881	34.8823
8	86.6768	54.5882	64.02	39.8655
9	97.6364	62.2855	72.1519	44.8487
10	108.596	69.9828	80.2838	49.8319
11	119.5556	77.6801	88.4157	54.8151
12	130.5152	85.3774	96.5476	59.7983
13	141.4748	92.0747	104.6795	64.7815

**Table 2:** The expected values of topological indices for $\delta _{1}=0$.

*k*	$E^{GA}$	$E^{ABC}$	$E^{ABS}$	$E^{R}$
4	42.7576	23.8797	31.5436	19.8989
5	53.6768	31.6174	39.7011	24.8652
6	64.596	39.3551	47.8586	29.8315
7	75.5152	47.0928	56.0161	34.7978
8	86.4344	54.8305	64.1736	39.7641
9	97.3536	62.5682	72.3311	44.7304
10	108.2728	70.3059	80.4886	49.6967
11	119.192	78.0436	88.6461	54.663
12	130.1112	85.7813	96.8036	59.6293
13	141.0304	93.519	104.9611	64.5956

## Comparison between the expected values of topological descriptors

**Figure 6:**
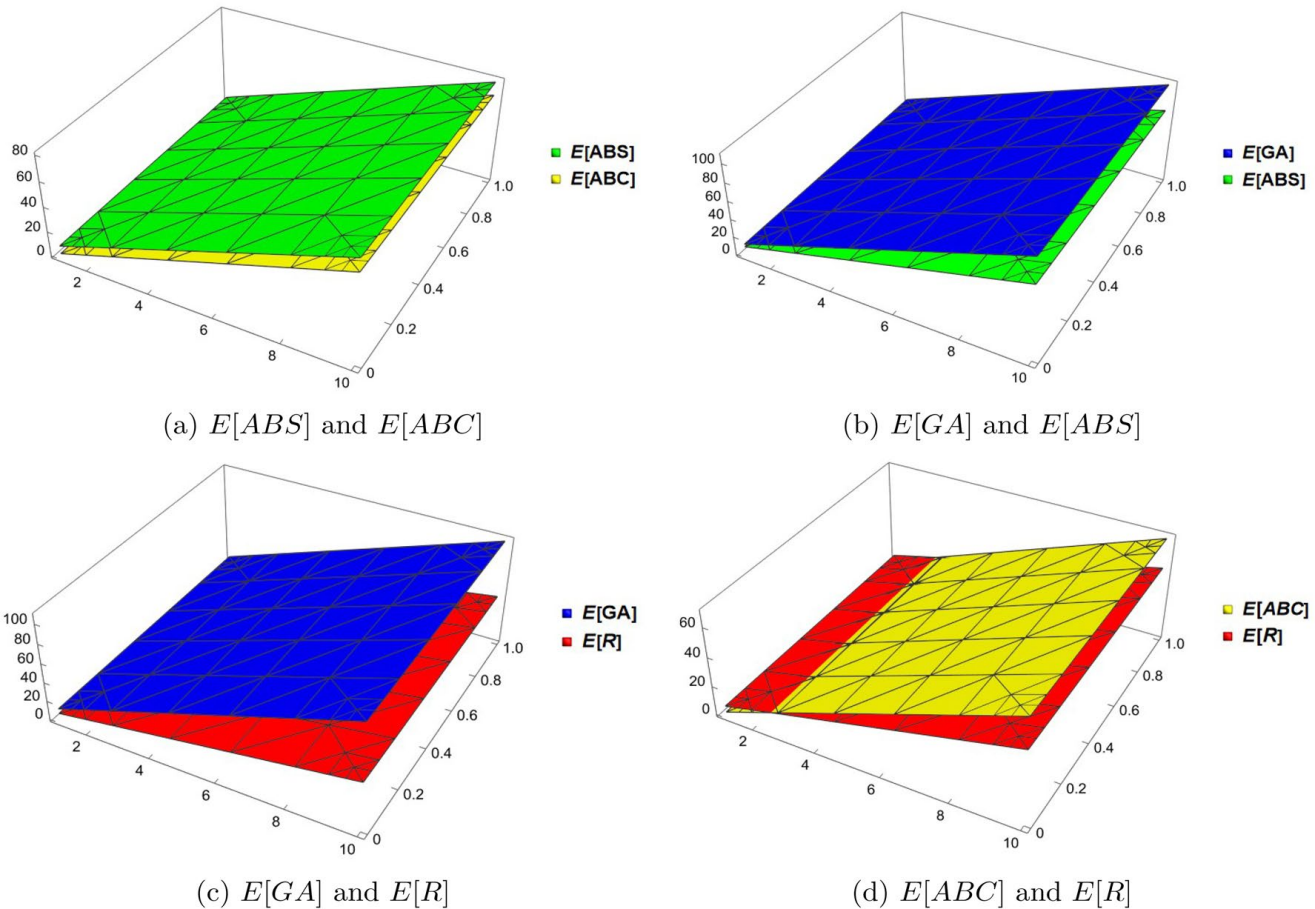
Graphical comparison between the expected values of topological indices.

**Figure 7:**
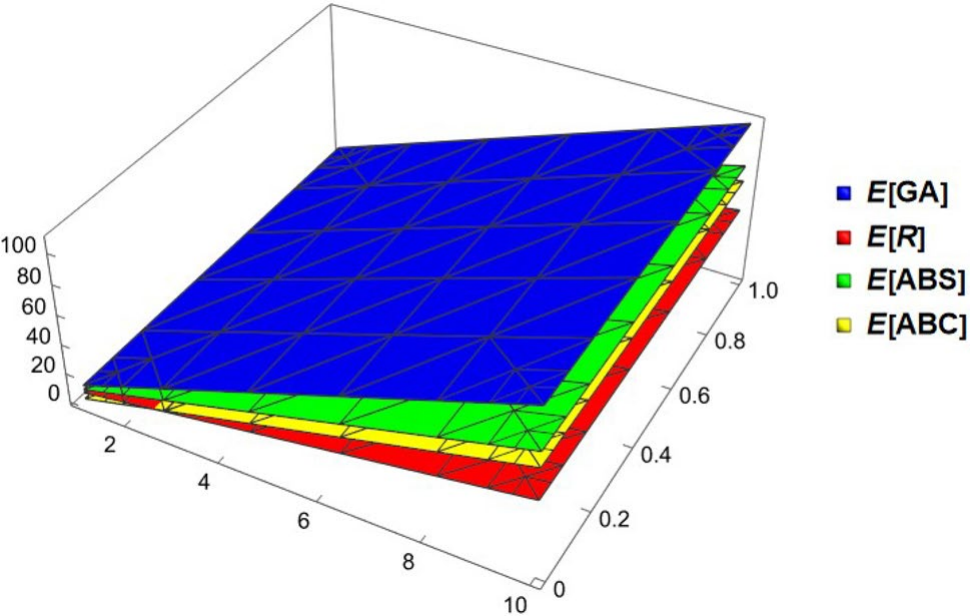
3D plots of *E*[*GA*], *E*[*ABS*], *E*[*ABC*] and *E*[*R*].

In this section we compare the expected values for the Randić, general Randić, atom-bound connectivity, atom-bound-sum connectivity and geometric-arithmetic indices for random cyclodecane chain having same probabilities. Tables 1, 2, 3, and 4 provides the numerical values of the expected values of these topological descriptors for different values of the probability function $\delta _{1}$. It is easy to observe that the value of geometric-arithmetic index is always greater than the other topological descriptors in all the cases. The comparison of the expected values of these topological descriptors can be seen in Figs. 6 and 7. Now, we give an analytical proofs for the comparison of the expected values of the considered topological descriptors.

**Table 3:** The expected values of topological indices for $\delta _{1}=1/2$.

*k*	$E^{GA}$	$E^{ABC}$	$E^{ABS}$	$E^{R}$
4	42.798	23.83935	31.518	19.9158
5	53.7374	31.55685	39.6627	24.89055
6	64.6768	39.27435	47.8074	29.8653
7	75.6162	46.99185	55.9521	34.84005
8	86.5556	54.70935	64.0968	39.8148
9	97.495	62.42685	72.2415	44.78955
10	108.4344	70.14435	80.3862	49.7643
11	119.3738	77.86185	88.5309	54.73905
12	130.3132	85.57935	96.6756	59.7138
13	141.2526	93.29685	104.8203	64.68855

**Table 4:** The expected values of topological indices for $\delta _{1}=1/4$.

*k*	$E^{GA}$	$E^{ABC}$	$E^{ABS}$	$E^{R}$
4	42.7778	23.85952	31.5308	19.90735
5	53.7071	31.587125	39.6819	24.877875
6	64.6364	39.314725	47.833	29.8484
7	75.5657	47.041325	55.9841	34.818925
8	86.495	54.769925	64.1352	39.78945
9	97.4243	62.497525	72.2863	44.759975
10	108.3536	70.225125	80.4374	49.7305
11	119.2829	77.952725	88.5885	54.701025
12	130.2122	85.680325	96.7396	59.67155
13	141.1415	93.407925	104.8907	64.642075

### Theorem 6

If $$k\ge 2$$, then $$ E[ABS(\mathbb {CDC}_k)] > E[ABC(\mathbb {CDC}_k)]$$.

### Proof

The statement is true for $k=2$. Now, we prove that the statement is true for $k>2$. By using Theorems 2 and 3, we have

$$\begin{aligned} E[ABS(\mathbb {CDC}_k)]-E[ABC(\mathbb {CDC}_k)]= & {} (k)(8.1575-0.0256\delta _{1})+0.0512\delta _{1}-1.0864\\{} & {} \displaystyle - (k)(7.7377-0.0404\delta _{1})-0.0809\delta _{1}+7.0711 \\= & {} (k)(0.4198+0.0148\delta _{1})-0.0297\delta _{1}+5.9847>0. \end{aligned}$$

$$\square $$

### Theorem 8

If $$k\ge 2$$, then $$E[GA(\mathbb {CDC}_k)] > E[R(\mathbb {CDC}_k)]$$.

### Proof

The statement is true for $k=2$. Now, we prove that the statement is true for $k>2$. By using Theorem 1 and 4, we have

$$\begin{aligned} E[GA(\mathbb {CDC}_k)]-E[R(\mathbb {CDC}_k)]= & {} (k)[(0.0404\delta _{1}+10.9192)]-0.0808\delta _{1}-0.9192\\{} & {} \displaystyle - (k)[(0.0169\delta _{1}+4.9663)]+0.0338\delta _{1}-0.0337 \\= & {} (k)(5.9529+0.0235\delta _{1})+0.1146\delta _{1}-0.9529>0. \end{aligned}$$

$$\square $$

### Theorem 9

If $$k\ge 2$$, then $$E[GA(\mathbb {CDC}_k)] > E[ABS(\mathbb {CDC}_k)]$$

### Proof

The statement is true for $k=2$. Now, we prove that the statement is true for $k>2$. By using Theorem 1 and 3, we have

$$\begin{aligned} E[GA(\mathbb {CDC}_k)]-E[ABS(\mathbb {CDC}_k)]= & {} (k)[(0.0404\delta _{1}+10.9192)]-0.0808\delta _{1}-0.9192\\{} & {} \displaystyle - (k)(8.1575-0.0256\delta _{1})-0.0512\delta _{1}+1.0864 \\= & {} (k)(2.7617+0.066\delta _{1})-0.132\delta _{1}+0.1672. \end{aligned}$$

$$\square $$

### Theorem 10

If $$k\ge 2$$, then $$E[ABC(\mathbb {CDC}_k)] > E[R(\mathbb {CDC}_k)]$$

### Proof

The statement is true for $k=2$. Now, we prove that the statement is true for $k>2$. By using Theorem 2 and 4, we have

$$\begin{aligned} E[ABC(\mathbb {CDC}_k)]-E[R(\mathbb {CDC}_k)]= & {} (k)(7.7377-0.0404\delta _{1})+0.0809\delta _{1}-7.0711\\{} & {} \displaystyle - (k)[(0.0169\delta _{1}+4.9663)]+0.0338\delta _{1}-0.0337 \\= & {} (k)(2.7714-0.0573\delta _{1})+0.1147\delta _{1}-7.1048. \end{aligned}$$

$$\square $$

### Corollary 11

If $$k\ge 2$$, then $$E[GA(\mathbb {CDC}_k)]> E[ABS(\mathbb {CDC}_k)]> E[ABC(\mathbb {CDC}_k)] > E[R(\mathbb {CDC}_k)]$$

### Proof

The result follows from Theorem 7, 8, 9 and 10. $\square$

## Conclusion

In this research, the expected values of Randić index, general Randić index, atom-bound connectivity index, atom-bound-sum connectivity index and geometric-arithmetic index for a random cyclodecane chain are computed and analyzed. Along with numerical and graphical representations of these indices in random cyclodecane chains, we also provide analytical proofs for comparisons indicating that the geometric-arithmetic index has the highest expected value of the other three topological indices.

## Data Availability

All data generated or analysed during this study are included in this published article.
